# Recombinant Integrin β1 Signal Peptide Blocks Gliosis Induced by Aβ Oligomers

**DOI:** 10.3390/ijms23105747

**Published:** 2022-05-20

**Authors:** Carolina Ortiz-Sanz, Francisco Llavero, Jone Zuazo-Ibarra, Uxue Balantzategi, Tania Quintela-López, Ane Wyssenbach, Estibaliz Capetillo-Zarate, Carlos Matute, Elena Alberdi, José L. Zugaza

**Affiliations:** 1 Achucarro Basque Center for Neuroscience, Science Park of the UPV/EHU, Sede Building, 3rd Floor, Barrio de Sarriena s/n, 48940 Leioa, Spain; carolinaortizsanz@gmail.com (C.O.-S.); francisco.llavero@ehu.eus (F.L.); jzuazoibarra@gmail.com (J.Z.-I.); uxue.balantzategi@ehu.eus (U.B.); t.lopez@ucl.ac.uk (T.Q.-L.); ane.wyssenbach@gmail.com (A.W.); estibaliz.capetillo@ehu.eus (E.C.-Z.); carlos.matute@ehu.eus (C.M.); 2 Department of Neurosciences, Faculty of Medicine and Nursery UPV/EHU and CIBERNED, Barrio de Sarriena s/n, 48940 Leioa, Spain; 3 IKERBASQUE, Basque Foundation for Science, Plaza Euskadi 5, 48009 Bilbao, Spain; 4 Department of Genetics, Physical Anthropology and Animal Physiology, Faculty of Science and Technology, UPV/EHU, Barrio de Sarriena s/n, 48940 Leioa, Spain

**Keywords:** Aβ oligomers, integrin β1, interactive region, astrogliosis, microgliosis, interferent peptides

## Abstract

Glial cells participate actively in the early cognitive decline in Alzheimer’s disease (AD) pathology. In fact, recent studies have found molecular and functional abnormalities in astrocytes and microglia in both animal models and brains of patients suffering from this pathology. In this regard, reactive gliosis intimately associated with amyloid plaques has become a pathological hallmark of AD. A recent study from our laboratory reports that astrocyte reactivity is caused by a direct interaction between amyloid beta (Aβ) oligomers and integrin β1. Here, we have generated four recombinant peptides including the extracellular domain of integrin β1, and evaluated their capacity both to bind in vitro to Aβ oligomers and to prevent in vivo Aβ oligomer-induced gliosis and endoplasmic reticulum stress. We have identified the minimal region of integrin β1 that binds to Aβ oligomers. This region is called signal peptide and corresponds to the first 20 amino acids of the integrin β1 N-terminal domain. This recombinant integrin β1 signal peptide prevented Aβ oligomer-induced ROS generation in primary astrocyte cultures. Furthermore, we carried out intrahippocampal injection in adult mice of recombinant integrin β1 signal peptide combined with or without Aβ oligomers and we evaluated by immunohistochemistry both astrogliosis and microgliosis as well as endoplasmic reticulum stress. The results show that recombinant integrin β1 signal peptide precluded both astrogliosis and microgliosis and endoplasmic reticulum stress mediated by Aβ oligomers in vivo. We have developed a molecular tool that blocks the activation of the molecular cascade that mediates gliosis via Aβ oligomer/integrin β1 signaling.

## 1. Introduction 

Alzheimer’s disease (AD) is the most common form of dementia and the most prevalent neurodegenerative disease [1]. Given that the first description made by Alois Alzheimer about pre-senile dementia refers to the formation of senile amyloid plaques and neurofibrillary tangles (aggregates of hyperphosphorylated tau protein) these elements are key pathological hallmarks of AD [2,3,4,5,6,7]. The formation of neurofibrillary tangles follows well-established patterns, while senile plaques appear and distribute in a random manner. The predictable alteration in the pattern and severity of the pathology permits the distinction of initial, intermediate and advanced stages based on investigations carried out by Braak and Braak [7]. In addition to plaque distribution, the detection of amyloid β (Aβ) as a main constituent of the plaques [8] and the identification of gene mutations related to Aβ synthesis in familial AD have led to formulating the amyloid cascade hypothesis [9,10], which postulates that Aβ deposition in the extracellular space leads to neurodegeneration and subsequent cognitive impairment [11,12,13]. This hypothesis is not only supported by autosomal-dominant Alzheimer’s disease (ADAD) but also an increase in the copy number of APP (e.g., triplication) is sufficient to cause AD and other amyloidosis [14]. However, the early CNS inflammation that aggravates the disease starts decades before the onset of AD, and it is characterized by neuronal and microglia-derived cytokines and chemokines, as well as mobilization of microglia toward Aβ-laden neurons [15].

Aβ peptide oligomers have been isolated from both animal models of AD [16,17] and cerebrospinal fluid (CSF) or brains from AD patients [18], in whom the presence of this peptide seems to correlate with the progression of the disease [19]. At nanomolar concentrations, Aβ oligomers are able to induce neuronal death in hippocampal organotypic slices [20,21], but also to inhibit long-term potentiation [21,22], and to promote abnormal Ca^2+^ fluxes as well as cell membrane disruption [20,23]. The biochemical and structural complexity of Aβ peptides make them very promiscuous molecules able to transduce signals through a repertoire of several receptors and proteins localized at the plasma membrane level both in neurons and in other cell types including glial cells [24,25].

Within the wide variety of effector molecules that interact with Aβ peptides, integrins have emerged as key molecules in the development of AD [25] by regulating synaptic dysfunction, diversity of plasticity and long-term potentiation in the early stages of neurodegenerative diseases [26]. Alpha v integrins mediate Aβ-induced inhibition of long-term potentiation [26]. Integrins are a complex family of glycoprotein receptors expressed ubiquitously [27,28]. In turn, they are a class of cellular adhesion molecules with adhesive and signal transduction functions [29,30] that drive to vital cellular events such as cell adhesion, differentiation or migration [31]. From a structural point of view, integrins are heterodimers constituted by alpha (α) and beta (β) subunits and bind non-covalently to mediate cell–cell and cell–extracellular matrix interactions. Each integrin recognizes specific ligands, which are either molecules of the extracellular matrix (ECM) (e.g., laminin and fibronectin) or other cell surface counter-receptors of the immunoglobulin superfamily (e.g., intracellular adhesion molecule-1 (ICAM-1)). However, integrins also have functional relationships with other membrane receptors such as ion channels including NMDA receptors and growth factor receptors [32].

During integrin activation, these glycoproteins change configuration from an inactive into an active form (stable extended high-affinity conformation) [33]. The active form triggers intracellular signaling cascades that are important for relaying information from the external environment to the inside of the cell. One of them is related to clustering between integrin and focal adhesions, leading to the assembly of numerous integrin-associated molecules such as talin, vinculin, paxillin, focal adhesion kinase (FAK), Src and integrin-linked kinase (ILK), that initiate canonical signaling pathways involving small GTPases of the Ras superfamily, ERK, JNK or AKT [34]. The heterodimers α2β1, α5β1, ανβ1 and ανβ3 can facilitate the deposition of Aβ and induce neurotoxicity, which results in neuronal loss [35,36,37]. However, the molecular mechanisms by which integrins participate in the development of AD are still unknown.

Here, we have mapped the extracellular region of integrin β1 in order to identify which domain binds to Aβ oligomers. Using an in vitro binding assay, we have revealed that Aβ oligomers bind to integrin β1 signal peptide localized at the first 20 amino acids (aa) at the N-terminal (hereinafter referred as R_s_). Application of this recombinant peptide in primary cultures of astrocytes inhibits ROS generation by Aβ oligomers. Moreover, we have analyzed in vivo the effects of the R_s_ peptide in Aβ oligomer-mediated astrogliosis and microgliosis, and in endoplasmic reticulum stress by performing intrahippocampal injections in mice. The findings reveal that integrin β1 signal peptide, R_s_, prevents gliosis and endoplasmic reticulum stress induced by Aβ oligomers in mouse hippocampus. Together, these data show that R_s_ peptide diminish Aβ oligomer-induced gliosis by interfering with integrin β1 signaling.

## 2. Results

### 2.1. Integrin β1 Signal Peptide Specifically Binds to Aβ Oligomers

**Figure 1:**
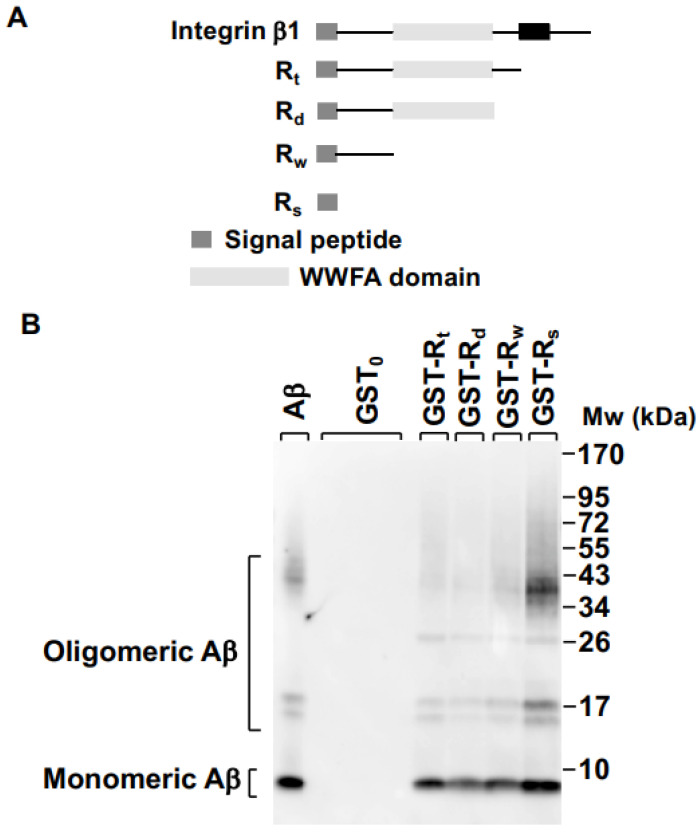

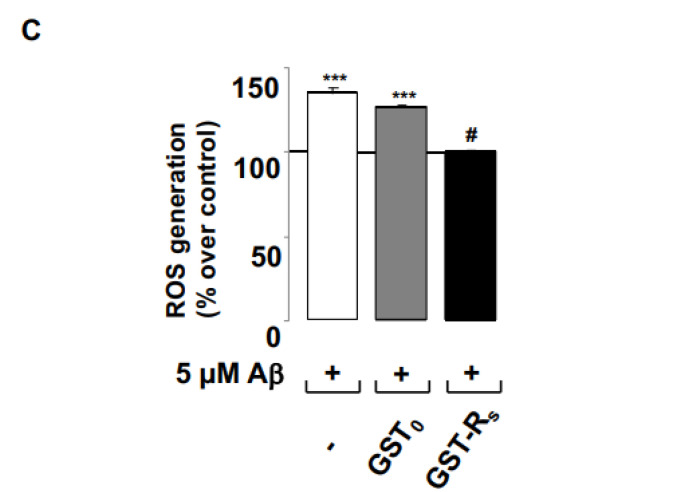
R_s_ peptide, carrying the signal peptide of integrin β1, specifically binds to Aβ peptide and blocks Aβ-induced ROS production in primary astrocyte cultures. (**A**) Schematic representation of the structure of integrin β1with its different extracellular regions. (**B**) Interaction of synthetic Aβ peptide with the indicated GST fusion proteins, GST_0_, GST-R_t_, GST-R_d_, GST-R_w_, and GST-R_t_. After incubation, glutathione beads were washed and proteins separated by SDS-PAGE under non-reducing conditions and analyzed by Western blot using anti-Aβ1–42 antibody (6E10, from Covance). (**C**) ROS generation was measured by fluorimetry with 10 µM CM-H2DCFDA. Data are expressed as the relative fluorescence normalized to values of untreated or treated cells (100%). *** *p* < 0.001 compared to non-treated cells; # *p* < 0.05 compared to GST_0_; unpaired one-way ANOVA.

First, we analyzed the amino acid sequence of integrin β1 and selected four regions from its extracellular domain. The first region was constituted by the first 20 amino acids (aa) and it was identified as R_s_, the second one, R_w_, included up to aa 139, the third region covered aa 1 to 378 including the VWA domain (R_d_), and the last region included the whole extracellular domain (from aa 1 to 728, R_t_) (Figure 1A). Now, to determine what amino acid stretch could represent an effective binding domain for Aβ oligomers, four recombinant GST fusion proteins were generated (R_s_, R_d_, R_w_, and R_t_, fused to the GST protein), and their binding capacities to Aβ oligomers were determined by affinity chromatography as described in the Experimental procedures section. As shown in Figure 1B, the four fused proteins bound not only to the monomeric Aβ peptide (the most intense band) but also to the oligomeric forms, the strongest interaction being between the oligomeric forms with the GST-R_s_ recombinant fusion protein (Figure 1B, lane 7). On the other hand, in order to verify that GST protein (GST_0_) was not involved in the interaction between Aβ and the fused proteins GST-R_s_, GST-R_d_, GST-R_w_, and GST-R_t_, we examined this possibility by affinity chromatography. As shown in Figure 1B (lane 2), GST_0_ had no ability to bind either monomeric or oligomeric Aβ. Figure 1B (lane 1) represents the reconstitution of synthetic Aβ (as an internal control) in its different forms visualized by Western blotting. Together, these findings identified that the signal peptide (R_s_) of the extracellular domain of integrin β1 was responsible for binding to Aβ oligomers in vitro. 

### 2.2. R_s_ Peptide Blocks Aβ Oligomer-Induced ROS Generation in Cultured Astrocytes 

Next, we investigated whether GST-R_s_ affected ROS generation mediated by Aβ oligomers in primary astrocyte cultures, as previously shown [38]. For that, we treated primary astrocyte cultures with 5 µM Aβ oligomers for 60 min alone or together with 5 µg/µL GST_0_ (control) or 5 µg/µL GST-R_s_, and measured ROS levels by fluorimetry using 10 µM CM-H2DCFDA for 20 min. As expected, Aβ oligomers induced ROS generation (Figure 1C, empty bar). Regarding GST_0_, this peptide did not interfere in Aβ oligomer-mediated ROS generation (Figure 1C, gray bar). Nevertheless, GST-R_s_ totally prevented ROS generation mediated by Aβ oligomers (Figure 1C, solid bar). Taken together, these results show that integrin β1 signal peptide (R_s_) binds in vitro to Aβ oligomers, and that it is able to prevent ROS generation induced by Aβ oligomers in primary astrocyte cultures.

### 2.3. Aβ Oligomers Trigger Gliosis in Mouse Hippocampus In Vivo

**Figure 2:**
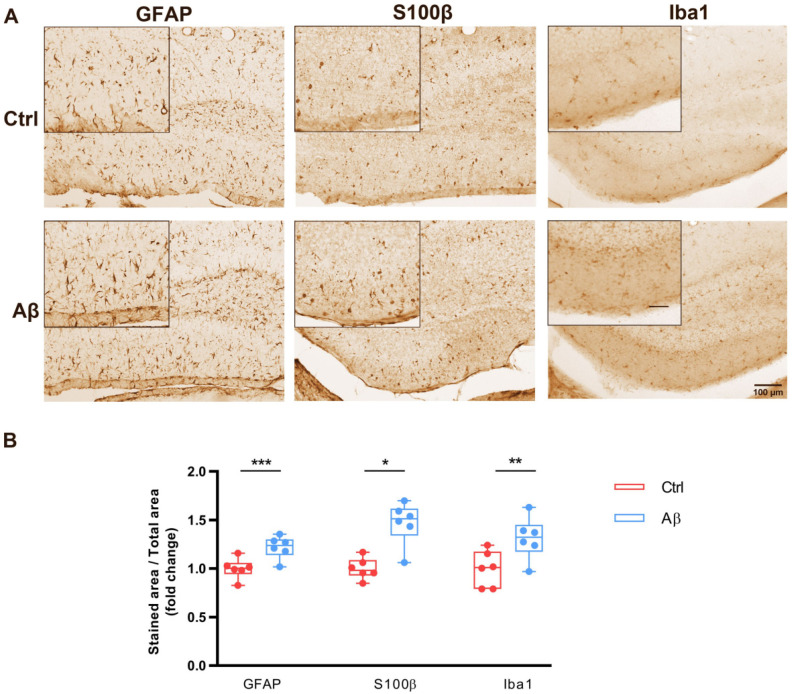
Reactive astrocytes and microglia in the dentate gyrus (DG) of Aβ-injected mice. (**A**) Coronal sections of mouse brains were immunostained by DAB assay 7 days post -injection with Aβ or with vehicle (Ctrl). Photomicrographs show GFAP and S100β immunolabeling in astrocytes and Iba1 immunolabeling in microglia of the dentate gyrus. Scale bar: 100 µm and Scale bar in zoom is 50 µm. It is included in caption.Inset: 50 µm. (**B**) Box plot graphs show quantitative analysis of labelled areas for GFAP, S100β and Iba1 under Aβ and control conditions in the DG. Data are presented as the mean ± S.E.M. Fifteen slices from five animals were analyzed per condition. *** *p* < 0.001, ** *p* < 0.01, * *p* < 0.05 compared with Aβ-injected mice; unpaired Student’s test.

Aβ injection in mouse brain causes reactive astrogliosis in the dentate gyrus (DG) [38]. However, it is still unclear whether Aβ injection in mice brain also drives microgliosis. To investigate that possibility, we performed intrahippocampal injections of vehicle (control) or Aβ oligomers (Aβ) and examined astrocyte- and microglia-occupied areas by immunohistochemistry with astrocyte (GFAP and S100β) and microglia (Iba1) markers in dentate gyrus (DG). As expected, the intrahippocampal administration of Aβ strongly increased the presence of both the GFAP and S100β markers compared to control (Figure 2A). In addition, Aβ also boosted the presence of the Iba1 marker in DG compared to control (Figure 2A). Quantification of the immunohistochemical analysis showed significant increases in the GFAP, S100β and Iba1 markers in DG values due to Aβ treatment compared to control (Figure 2B, 1.00 ± 0.04 vs. 1.21 ± 0.04 for GFAP, 1.00 ± 0.04 vs. 1.46 ± 0.08 for S100β 1.00 ± 0.07 vs. 1.31 ± 0.08 for Iba1). These results confirm that Aβ induces astrogliosis and show that Aβ oligomers also lead to microgliosis in adult mouse DG.

### 2.4. R_s_ Peptide Prevents Glia Reactivity in the DG of Aβ Oligomer-Injected Mice Brain

**Figure 3:**
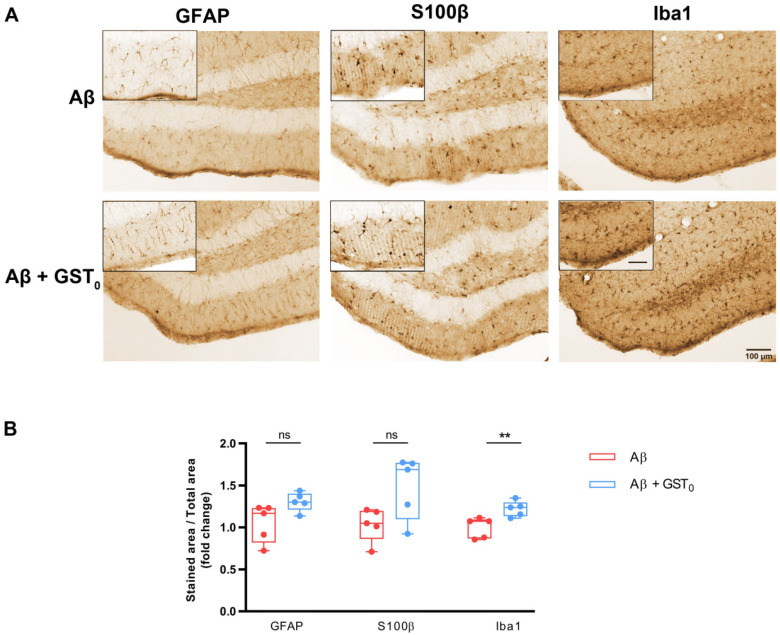
GST_0_ polypeptide is ineffective in preventing gliosis in the DG of Aβ-injected mice. (**A**) Coronal sections of mouse brains were immunostained by DAB assay 7 days post-injection with Aβ and Aβ + GST_0_. Photomicrographs show GFAP and S100β immunolabeling in astrocytes and Iba1 immunolabeling in microglia of the dentate gyrus. Scale bar: 100 µm and Scale bar in zoom is 50 µm. It is included in caption: 50 µm. (**B**) Box plot graphs show quantitative analysis of labelled areas for GFAP, S100β and Iba1 under Aβ and Aβ + GST_0_ in the DG. Data are presented as the mean ± S.E.M. Fifteen slices from five animals were analyzed per condition. ns: non-significant; ** *p* < 0.01 compared with Aβ-injected mice; unpaired Student’s test.

Before examining the functionality of the GST-R_s_ fused protein in vivo, we evaluated whether GST_0_ affected astrocyte and microglia reactivity in Aβ oligomer-injected brain. For that, we performed intrahippocampal injections of Aβ and Aβ with GST_0_ (Aβ + GST_0_) and quantified the changes in astrocyte and microglia morphology as described in the previous section. As shown in Figure 3A, the intrahippocampal administration of the combination of Aβ + GST_0_ did not modify the area occupied by both the GFAP and S100β markers compared to Aβ alone. However, the area occupied by Iba1 staining appeared increased in the combination Aβ + GST_0_ when it was compared to Aβ alone (Figure 3A). Quantification of the immunohistochemical analysis showed that GST_0_ in the presence of Aβ did not produce any significant change in GFAP and S100β staining (Figure 3B; 0.94 ± 0.09 vs. 0.64 ± 0.03 for GFAP, 0.93 ± 0.08 vs. 0.67 ± 0.06 for S100β, whereas it caused microgliosis as compared to Aβ alone (1.00 ± 0.04 vs. 0.77 ± 0.03 for Iba1). These results suggest that the GST_0_ protein did not reduce Aβ-dependent astrogliosis and/or microgliosis.

**Figure 4:**
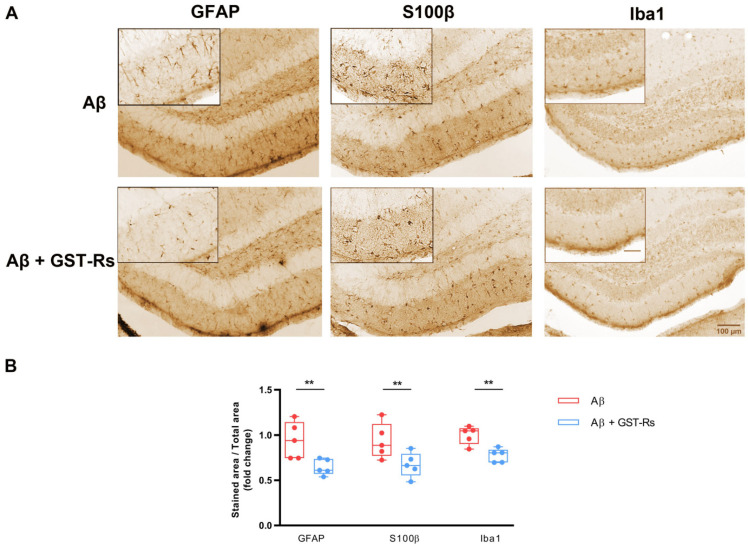
GST-R_s_ polypeptide prevents gliosis in the DG of Aβ-injected mice. (**A**) Coronal sections of mouse brains were immunostained by DAB assay 7 days post-injection with Aβ or Aβ + GST-Rs. Photomicrographs show GFAP and S100β immunolabeling in astrocytes and Iba1 immunolabeling in microglia of the dentate gyrus. Scale bar: 100 µm and Scale bar in zoom is 50 µm. It is included in caption. 50 µm (**B**) Box plot graphs show quantitative analysis of labelled areas for GFAP, S100β and Iba1 under Aβ and Aβ + GST-R_s_ in the DG. Data are presented as the mean ± S.E.M. Fifteen slices from five animals were analyzed per condition. ** *p* < 0.01 compared with Aβ-injected mice; unpaired Student’s test.

Based on that, we examined the ability of recombinant GST-R_s_ peptide to prevent Aβ-mediated astrogliosis in brain. For that, we performed intrahippocampal injections of Aβ, and Aβ with GST-R_s_ peptide (Aβ + GST-R_s_) and the glial changes were analyzed and quantified. As shown in Figure 4A, the intrahippocampal administration of the combination of Aβ + GST-R_s_ strongly reduced the presence of three—GFAP, S100β and Iba1—markers compared to Aβ.

Quantification of the immunohistochemical analysis showed a significant decrease in GFAP, S100β and Iba 1 (Figure 4B) in the presence of Aβ + GST-R_s_ compared to Aβ (1.05 ± 0.10 vs. 1.30 ± 0.05 for GFAP, 1.03 ± 0.08 vs. 1.484 ± 0.167 for S100β 1.00 ± 0.05 vs. 1.22 ± 0.04 for Iba1). These results point out that integrin β1 signal peptide R_s_ blocks Aβ-induced not only astrogliosis but also in microgliosis in adult mouse DG.

### 2.5. R_s_ Peptide Reduces Endoplasmic Reticulum Stress in Astrocytes in DG of Aβ Oligomer-Injected Mice Brain

**Figure 5:**
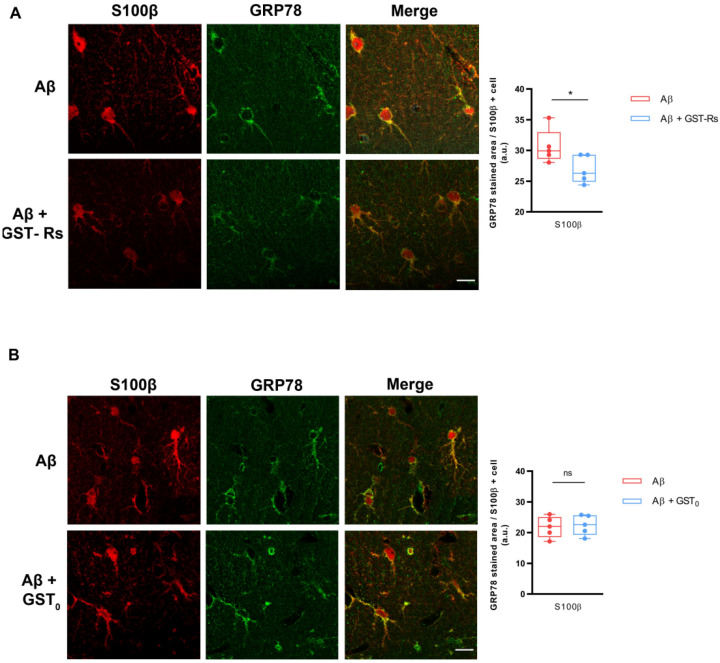
GST-R_s_ polypeptide reduces GRP78 expression in S100β-positive astrocytes of Aβ-injected mouse brains. Photomicrographs of double immunofluorescence staining for S100β (red) and GRP78 (green) on DG of animals injected with different: Aβ and Aβ + GST-R_s_ (**A**) or Aβ and Aβ + GST_0_ (**B**). Quantitative analysis of fluorescence intensity was performed for GRP78 levels in S100β-positive astrocytes in dentate gyrus after Aβ and Aβ + GST-Rs (**A**) or Aβ and Aβ + GST_0_ (**B**). Scale bar in zoom area: 20 µm. Data are presented as the mean ± SEM. Fifteen slices from five animals were analyzed per condition. ns: non-significant; * *p* < 0.05 compared with Aβ-injected mouse; unpaired Student’s test.

Acute injection of Aβ oligomers in mouse brain induces GRP78 chaperone protein overexpression particularly in astrocytes [39], being used as an endoplasmic reticulum stress marker. Therefore, we investigated whether recombinant R_s_ fused protein to GST (GST-R_s_) could also prevent endoplasmic reticulum stress in astrocytes after intrahippocampal Aβ injection. Accordingly, we carried out a double immunostaining assay for S100β and GRP78 of brain tissues previously injected with Aβ, Aβ + GST-R_s_ and Aβ + GST_0_. Intrahippocampal administration of the combination of recombinant GST-R_s_ peptide and Aβ oligomers strongly reduced GRP78 expression in S100β-positive astrocytes compared to Aβ oligomers alone (Figure 5A,B). Furthermore, the combination of GST_0_ and Aβ oligomers did not alter the effect induced by Aβ alone (Figure 5B). Quantification of immunofluorescence staining showed a significant decrease in GRP78 in S100β values in DG from brains injected with GST-R_s_ fusion protein compared to control (Aβ-injected mice) (26.95 ± 1.01 vs. 30.64 ± 1.24) (Figure 5A). In contrast, GST_0_ protein did not produce any effect in Aβ-induced endoplasmic reticulum stress in S100β (Figure 5B) values compared to Aβ alone (21.42 ± 2.48 vs. 22.07 ± 1.36). These findings suggest that R_s_ also prevents endoplasmic reticulum stress induced by Aβ oligomers. 

## 3. Discussion

Our study identifies the integrin β1 minimal region that binds to Aβ oligomers. This region spans from aa 1 to aa 20 and corresponds to integrin β1 signal peptide (R_s_ peptide). From a functional point of view, this peptide is a very useful tool to block Aβ oligomer-induced ROS generation in primary astrocyte cultures and also in vivo when R_s_ peptide in combination with Aβ oligomers is directly injected into the mice hippocampus. In this scenario, astroglial stress, astrogliosis and even microgliosis induced by Aβ oligomers are efficiently prevented. 

Several investigations postulate that there are many potential receptors localized at neuronal synapses with both high affinity for Aβ peptide and the ability to intracellularly transduce the toxic instructions emanating from Aβ oligomers [40]. These include NMDA receptors that are directly activated by Aβ oligomers, altering its physiological function [41], although those that seem to be acquiring increasing relevance are integrins. In fact, the interaction between integrins and Aβ oligomers promotes neurotoxicity, inhibition of LTP and an increase in spine density [26,42]. In this regard, synthetic Aβ monomer binds through its amino acid sequence RHDS to the α2bβ3 integrin, being directly related to cerebral amyloid angiopathy, which contributes to dementia and AD [43]. 

Integrins control important cellular responses including proliferation, survival and cell migration [44]. All of them require the active participation of transducing molecules such as tyrosine kinases FAK, ILK and Src or small GTPases of the Rho family [44]. In addition, PKCs may also be involved in integrin-mediated signaling [45]. We have previously observed that Aβ oligomer-induced PKC phosphorylation is mediated by integrin β1 in astrocytes and in neurons [38]. Further, Aβ oligomers lead to NR2B subunit upregulation on neuronal membranes through the PKC signaling pathway [46]. Under these circumstances, integrin β1 transduces the message that Aβ oligomers brings, generating a cellular response which manifests itself in a higher permeability for calcium ions to alter cellular homeostasis [46]. Hence, depending on the stimulus or ligands, the same receptor along with its intracellular signaling molecules can switch on/off different pathways that lead to antagonistic cellular responses. 

**Figure 6:**
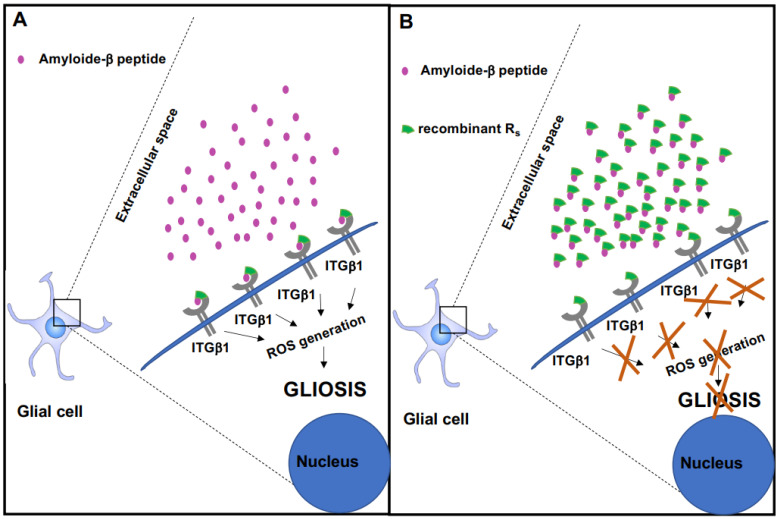
Functional model of recombinant Rs peptide to prevent the toxic effect from amyloide β peptide. (**A**) Amyloide β peptide from the extracellular space binds to integrin β1 and triggers the activation of intracellular signaling pathways that lead to the generation of reactive oxygen species and gliosis. (**B**) Recombinant R_s_ peptide and amyloide β peptide interact with each other; and in these circumstances, amyloide β peptide cannot bind to the R_s_ region of integrin β1 and does not activate the signaling pathways that lead to ROS generation and gliosis.

Currently, in addition to pharmacotherapy, gene therapeutic approaches for AD have entered phase I/II clinical trials [47]. The results of this preliminary study obtained with recombinant R_s_ allow us to postulate a new pharmacological therapeutic alternative in AD. This recombinant peptide neutralizes Aβ oligomer activity from outside the cell (Figure 6 panel B compared to panel A). In addition, Rs recombinant peptide is a useful tool that will aid understanding the molecular mechanisms of the deleterious actions initiated by Aβ oligomers both in vitro and in vivo. 

## 4. Conclusions

We and others have described a key molecular relationship between integrin β1 and Aβ peptides required to modulate neuronal and glial biology [27,38,42,46]. The findings point out the molecular mechanism by which recombinant R_s_ peptide works in order to block Aβ oligomer intracellular signaling both in vitro and in vivo. The presence of this recombinant peptide in the extracellular medium interferes with binding between Aβ oligomers and its receptor, integrin β1, since the R_s_ peptide associates with Aβ oligomers, thus preventing it from binding to the endogenous integrin β1, and consequently avoiding the transmission of its toxic message. In fact, R_s_ peptide blocks ROS generation induced by Aβ oligomers and at the same time significantly reduces astroglial stress, astrogliosis and microgliosis. It is important to highlight that Rs peptide in turn protects the functional receptorial properties of integrin β1, allowing integrin β1 in the cell membrane to be accessible to physiological activators (Figure 6). Future studies will allow us to investigate the efficacy of this peptide in preventing Aβ oligomers binding to other receptors.

## 5. Experimental Procedures

Animals. All experimental procedures (M20-2017-092) followed the European Directive 2010/63/EU and were approved by the ethics committee of the University of the Basque Country (UPV/EHU). Animals were housed in standard conditions under 12 h light/dark cycle and with ad libitum access to food water. All possible effort was made to minimize animal suffering and the number of animals used. Experiments were performed in C57BL6/J mice. 

Preparation of Aβ_1–42_ Oligomers. Aβ_1–42_ oligomers were prepared as reported previously [48]. Briefly, Aβ1–42 was initially dissolved to 1 mM in hexafluoroisopropanol (Merck Life Science S.L.U., Madrid, Spain) and distributed aliquoted in sterile microcentrifuge tubes. Hexafluoroisopropanol was totally removed under vacuum in a speed vac system and the peptide film was stored at −80 °C. For the aggregation protocol, the peptide was first resuspended in anhydrous DMSO (Merck Life Science S.L.U., Madrid, Spain) to a concentration of 5 mM, to finally bring the peptide to a final concentration of 100 μM in Hams F-12 (Merck Life Science S.L.U., Madrid, Spain and to incubate it at 4 °C for 24 h. The preparation was then centrifuged at 14,000× *g* for 10 min, at 4 °C, to remove insoluble aggregates and the supernatants containing soluble Aβ_1__–42_ were transferred to clean tubes and stored at 4 °C.

Plasmid Construct. The ITGβ1 fragments comprising amino acids 1–20 (R_s_), 1–140 (R_w_), 1-371 (R_d_) and 1–728 (R_t_) were generated by PCR amplification using pCMV6-XL5-ITGB1 (from Origene Technologies Inc. Rockville, MD, USA) as template (forward oligonucleotide, 5′-CGG AAT TCA TGA ATT TAC AAC C-3′ and reverse oligonucleotides, 5′-CGG AAT TCA GCA AAC ACA CAG C-3′, 5′-CGG AAT TCG TCT TCA GCT CTC T-3′, 5′-CGG AAT TCA AGG GAA TTG TAT G-3′, 5′-CGG AAT TCG TCT GGA CCA GTG G-3′, each harboring EcoRI restriction sites (underlined). The EcoRI ITGβ1 extracellular fragments were subcloned into pGEX-4T3 (Merck Life Science S.L.U., Madrid, Spain) to generate the GST-R_s_, GST-R_w_, GST-R_d_ and GST-R_t_ fusion proteins. All GST-fused peptides were purified by affinity chromatography onto glutathione beads following standard procedures [49].

Binding Assay. In vitro binding assays with recombinant fusion proteins were performed as previously described [50]. Briefly, glutathione beads coated with recombinant fusion proteins (500 ng GST_0_, GST-R_t_, GST-R_w_, GST-R_d_ or GST-R_s_) were incubated with 100 pM Aβ oligomers in binding buffer (50 mM Tris-HCl pH7.5, 5 mM MgCl_2_, 20 mM KCl, 500 µg/mL BSA) for 1 h at RT. Immobilized GST beads were washed twice with binding buffer and five times with 50 mM Tris-HCl pH 7.5, 150 mM NaCl. Proteins were eluted adding sample buffer under non-reducing conditions and separated by SDS-PAGE followed by Western blot. Immunoreactive bands were visualized with anti-6E10 antibody and ECL.

Astrocyte Culture. Primary cultures of cerebral cortical astrocytes were prepared from P_0_–P_2_ Sprague Dawley rats as previously described [51]. Cortical lobes were extracted and enzymatically digested with 400 μL of 2.5% trypsin and 40 μL of 0.5% deoxyribonuclease in Hank’s Balanced Salt Solution (HBSS, Merck Life Science S.L.U., Madrid, Spain) for 15 min at 37 °C. The enzymatic reaction was stopped by adding IMDM medium supplemented with 10% FBS (Thermo Fisher Scientific, Madrid, Spain) and centrifuged at 300× *g* for 6 min. The cell pellet was resuspended in 1 mL of the same solution and mechanical dissociation was performed by using 21- and 23G-gauge cutting needles. The resulting cell suspension was centrifuged at 300× *g* for 6 min and plated onto 75 cm^2^ flasks coated with 30 µg/mL Poly-D-Lysine. After 8 DIV, cells were plated onto PDL-coated plates and maintained for 2 days. The culture medium was replaced with IMDM with 1% FBS 24 h before Aβ treatment.

Measurement of Intracellular Reactive Oxygen Species. For the quantification of generated ROS in treated cells, fluorescent dye 5-(and 6)-chloromethyl-2′7dichlorodihydrofluorescein diacetate acetyl ester (CM-H2DCFDA) was used. Astrocytes (1 × 10^4^) were exposed to 5 µM Aβ oligomers [29,30] alone or together with 5 µg/µL GST_0_ or 5µg/µL GST-R_s_ and loaded with 10 μM CM-H2DCFDA for 30 min immediately after the treatment. After three washes with PBS, ROS levels were measured with excitation and emission wavelengths of 485 and 520 nm, respectively.

Intrahippocampal Injection in Adult Mice. Adult male mice (3–4 months) were randomized, anesthetized with ketamine hydrochloride (80 mg × kg^−1^) and xylazine (10 mg × kg^−1^), and injected stereotaxically into the hippocampus at the following coordinates: 2.2 mm from Bregma, 1.5 mm lateral to the sagittal suture, and 2 mm from the pial surface. Mice were divided into four groups (n = 5–6 per group) and injected with 3 µL of either vehicle (17% DMSO + 83% Ham’s F12; control), Aβ oligomers (10 μM; Aβ), Aβ plus GST_0_ (10 μM and 0.45 μg/μL, respectively; Aβ + GST_0_), or Aβ plus R_s_ peptide (10 μM and 0.45 μg/μL, respectively; Aβ + R_s_). After 7 days, mice were anesthetized with ketamine hydrochloride (80 mg × kg^−1^) and xylazine (10 mg × kg^−1^) and perfused with 30 mL of phosphate buffer followed by 30 mL of 4% PFA (paraformaldehyde) in 0.4 M PBS (pH 7.5). The brains were extracted and post-fixed with the same fixative solution for 4 h at RT, placed in 30% sucrose in 0.1 M PBS pH 7.5 at 4 °C, and then kept in cryoprotectant solution (30% ethylene glycol, 30% glycerol and 0.1 M PBS in dH_2_O) at −20 °C.

Brain Slice Preparation and Immunostaining. Brain tissue was cut using a Leica VT 1200S vibrating blade microtome (Leica microsystems). Coronal 40 μm thick sections were washed in PBS and incubated with 0.1 M PBS containing 3% H_2_O_2_ for 10 min at RT. Then, slices were rinsed three times with PBS and blocked in blocking solution (PBS pH 7.5, 4% HS, 0.1% Triton X-100) for 30 min at RT. Next, slices were incubated with the corresponding specific primary antibodies (rabbit anti-GFAP (1:1000 from Merck Life Science S.L.U., Madrid, Spain), rabbit anti-S100β (1:500 from Dako, Glostrup, Denmark) or rabbit anti-Iba1 (1:250 from Fujifilm Wako Chemicals, Richmond, VA, USA), in the same blocking solution, overnight at 4 °C with gentle shaking. Next, slices were washed three times with PBS and incubated with secondary antibodies (1:500 from Vector Laboratories Burlingame, CA, USA) in the blocking solution for 1 h at RT. Slices were incubated with the ABC complex following the manufacturer’s instructions (Vector Laboratories, Burlingame, CA, USA) for 1 h at RT and washed three times with PBS. Slices were treated with DAB (Vector Laboratories Burlingame, CA, USA) according to the manufacturer’s instructions and washed three times with PBS. Finally, slices were mounted on glass slides with DPX.

For immunofluorescence of brain slices, slices were kept in PBS at 4 °C and permeabilized and blocked with 0.1 M PBS pH 7.5, 10% NGS, and 0.1% Triton X-100 for 1 h at RT. Slices were incubated with primary antibodies (rabbit anti-S100β (1:500 from Dako, Glostrup, Denmark) and mouse anti-GRP78 (1:500 from Elabscience, Houston, TX, USA)) overnight at 4 °C with gentle shaking. Slices were then washed three times with 0.1 M PBS pH 7.5, 0.1% Triton X-100 (washing buffer) and incubated with blocking solution containing fluorochrome-conjugated secondary antibodies for 1 h at RT. After that, slices were washed three times with washing buffer, incubated with 4 μg/mL DAPI, washed twice again with washing buffer and mounted on glass slides with Fluoromount-G mounting medium (SouthernBiotech, Birmingham, AL, USA).

Image acquisition and analysis. Brightfield images were acquired with the Pannoramic MIDI II automated digital slide scanner (3DHistech Ltd., Budapest, Hungary). To analyze reactive gliosis, the area occupy by DAB divided by total area was measured.

Fluorescence immunostaining was observed with a Leica TCS SP8 microscope using a 63× oil-immersion objective to generate z-stack projections. For fluorescence intensity analysis, images were taken with the same settings for all experiment and the mean value along the stack profile was quantified with LAS AF Lite software, version 4.0, Leica Microsystems CMS GmbH, Shinjuku, Tokyo, Japan (Leica). 

Statistical analysis. All data were expressed as the mean ± S.E.M. Statistical analyses were performed using absolute values. GraphPad Prism software (https://www.graphpad.com/scientific-software/prism/, accessed on 1 April 2022) was used applying one-way analysis of variance with post hoc Fisher’s least significant difference (LSD) test for multiple comparisons and two-tailed, unpaired Student’s *t* test for comparison of the two groups and control conditions.

## Data Availability

The datasets generated and/or analyzed during the current study are available from the corresponding authors on reasonable request.

## Abbreviations

Aβ—amyloid β protein fragment 1–42; AD—Alzheimer’s disease; AKT—protein kinase B; CM-H2DCFDA—5-(and-6)-chloromethyl-2′,7′-dichlorodihydrofluorescein diacetate, acetyl ester; DAB—3, 3′-diaminobenzidine; CSF—cerebrospinal fluid; DAPI—4′,6-diamidino-2-phenylindole; DMSO—dimethyl sulfoxide; DPX—distyrene, a plasticizer and xylene; ECM—extracellular matrix; ERK—extracellular signal-regulated kinase; FAK—focal adhesion kinase; GFAP—glial fibrillary acid protein; GST—glutathione S-transferase; ILK—integrin-linked kinase; ITGβ1—integrin β1; JNK—c-Jun N-terminal kinase; NFT—neurofibrillary tangles; ROS—reactive oxygen species; ICAM-1—intracellular adhesion molecule-1; NMDA—N-methyl-D-aspartate.
